# Extraction of Kinematic Parameters and Comparative Study of Endurance Levels in Mongolian Horses

**DOI:** 10.3390/vetsci13040404

**Published:** 2026-04-20

**Authors:** Yakai Shen, Lide Su, Yong Zhang, Jin Liu, Zhihao Zhang, Shun Zhang

**Affiliations:** 1 College of Mechanical and Electrical Engineering, Inner Mongolia Agricultural University, Hohhot 010018, China; 15238249323@163.com (Y.S.);; 2 Full Mechanization Research Base of Dairy Farming Engineering and Equipment, Ministry of Agriculture and Rural Affairs of the People’s Republic of China, Hohhot 010018, China; 3 Inner Mongolia Engineering Research Center of Intelligent Equipment for the Entire Process of Forage and Feed Production, Hohhot 010018, China; 4 Modern Agriculture and Animal Husbandry Development Center, Bayannur, Inner Mongolia Autonomous Region, Bayannur 015001, China

**Keywords:** kinematic parameters, endurance-race performance, Mongolian horse, motion capture, skeletal model

## Abstract

Mongolian horses are an indigenous Chinese breed known for their endurance capacity, yet quantitative descriptions of their gait-related kinematic characteristics remain limited. This pilot exploratory study aimed to describe the kinematics of Mongolian horses during walk, slow trot, and fast trot, and to examine whether selected variables differed between race-result groups in a 12 km endurance race. Forty-six horses were classified into an excellent group and an ordinary group based on the result of a single race. Kinematic data were collected using optical motion capture and three-dimensional skeletal modelling. Separate gait-specific linear mixed-effects models were fitted, with horse identity as a random effect and group and speed as fixed effects. The results showed gait-dependent between-group differences. During walk, the excellent group had significantly greater range of motion of the tarsal, hip, and elbow joints, as well as a greater maximum forelimb retraction angle (all *p* < 0.001). During slow trot, the excellent group showed significantly greater stride length (*p* = 0.009), elbow joint range of motion (*p* < 0.001), minimum hindlimb forward extension angle (*p* = 0.033), and minimum forelimb forward extension angle (*p* = 0.004). During fast trot, the between-group differences were most pronounced, with significantly greater stride length (*p* < 0.001) and range of motion of the tarsal joint (*p* < 0.001), hip joint (*p* = 0.015), and elbow joint (*p* = 0.014), together with greater maximum hindlimb retraction angle (*p* = 0.001) and minimum forelimb forward extension angle (*p* = 0.026). Overall, these findings provide preliminary evidence that gait-related kinematic differences may exist between race-result groups in Mongolian horses. However, because this was an exploratory study based on a single race, the findings should be interpreted cautiously and require validation in larger and more diverse cohorts.

## Simple Summary

This pilot study preliminarily explored the kinematic characteristics of Mongolian horses with different endurance-race outcomes during walk, slow trot, and fast trot, and examined whether selected kinematic variables differed between performance groups. A total of 46 Mongolian horses were classified into an excellent group (*n* = 23) and an ordinary group (*n* = 23) based on their results in a 12 km endurance race. A motion capture system was used to record the spatial positions of 71 anatomical landmarks during the three gaits. Skeletal models were constructed using Visual3D to extract nine kinematic parameters under each locomotor condition. The results suggested that several kinematic variables showed gait-dependent differences between the two groups.

## 1. Introduction

The Inner Mongolia Autonomous Region is renowned as the home of the Mongolian horse and has a long history of equine breeding, rearing, and racing. It possesses profound cultural heritage and favourable geographical and climatic conditions for developing a modern equine industry [1]. In recent years, a modern equine industry has emerged in China. Closely related sectors—including equestrian sports, recreational riding, cultural tourism, and specialized product development—are flourishing and gaining consumer popularity [2]. However, China’s equine sector currently lags behind emerging development trends. Key shortcomings include outdated production methods, underutilization of the superior traits of local horse breeds, and poor integration with related sectors—such as equestrian sports, fitness, ethnic culture, and leisure tourism. Competition horses are predominantly imported, which, coupled with the underutilization of local horse breeds’ superior traits, results in an insufficient domestic supply of sport horses to meet market demand [3]. As an excellent local horse breed in China, the Mongolian horse is renowned for its extraordinary endurance and holds a unique competitive edge in long-distance endurance races, making it a core resource to solve the supply problem of domestic endurance horses.

Research into the endurance traits of Mongolian horses has largely focused on molecular biology. Key physiological foundations of their endurance superiority have been identified, including a high proportion of slow-twitch muscle fibres, enhanced fatty acid oxidation, low creatine kinase activity, enlarged heart volume, and a distinctive nasal structure [4]. In addition, Yang Lihua conducted molecular identification of candidate genes and proteomes related to the movement of Mongolian horses [5]. Wu Suguguga analyzed the expression and gene polymorphism of candidate genes for movement and Wei Ruiyuan explored the plasma metabolic characteristics of Mongolian horses with different endurance-race performance [6,7]. However, there are still few reports on the phenotypic parameters of Mongolian horses. The gait characteristics in phenotypic features are the core indicators affecting the movement performance of horses, and “gait quality” as a key factor for evaluating elite horses currently lacks an objective and quantitative measurement standard, especially a systematic study on the correlation between different endurance-race performance of Mongolian horses and gait characteristics, which makes it difficult to accurately screen individuals with high endurance potential through phenotypic indicators in breeding practice [8].

The quantification of gait characteristics relies on scientific detection techniques. Three main methods exist for equine gait analysis. The first, two-dimensional video-based motion capture software, is convenient but has relatively large errors [9]. The second, inertial measurement units (IMUs) as wearable sensors, offers key advantages—unrestricted spatial application and occlusion insensitivity—making it ideal for outdoor and real-time use [10]. However, inertial sensors have notable limitations in practice: they suffer from cumulative drift due to integration, are highly susceptible to magnetic interference, and require complex calibration—all of which can undermine long-term data reliability [11,12]. Optical motion capture systems, by contrast, track reflective markers via cameras to deliver high-precision absolute position data, supported by efficient processing software, and are established as the industry benchmark [13]. This technology has been maturely applied in other horse breed studies: Marin studied the movement parameters of Menorca horses during walking and trotting, clarifying the differences in limb movement compared to other dressage horse breeds [14]. Agneta Egenvall used optical motion capture equipment to analyze the back parameters of Iberian horses under different gaits [15]. In the correlation analysis of movement performance, Vicki A. Walker confirmed that increasing the range of joint movement can enhance the movement ability of horses [16]. Rhodin found through a mixed model that the decline in movement performance is related to the increase in asymmetry of the horse’s body and limb movement [17]. Zeng Yaqi et al. took 72 two-year-old Yili horses as the subjects and confirmed that the larger the stride index and the gait frequency index, the better the movement performance of the horse [18]. The formation and optimization of these core gait parameters essentially depend on the precise regulation of the joint angles of the limbs, highlighting the core value of joint angles in evaluating the movement ability of horses [19].

For veterinary clinical practice, the athletic performance of endurance racehorses is closely related to their health. During long-distance races, the joints of the horse’s limbs are subject to cumulative loads. Unreasonable gait characteristics can significantly increase the risk of joint injuries, tendinitis and other sports-related diseases, thereby shortening the horse’s athletic career. The core significance of conducting a comparative study on the gait characteristics of Mongolian horses with different endurance-race performance lies in providing a “non-invasive” assessment tool for the movement function in veterinary clinical practice: by clarifying key differences in gait parameters between the excellent group and the ordinary group, this study may provide preliminary reference information for future research on performance-related phenotyping, individualized training strategies, and rehabilitation monitoring in endurance horses [20].

In general, current studies on Mongolian horses lack a systematic analysis of gait-related kinematic features in relation to performance in endurance competitions. This pilot study aimed to preliminarily describe the kinematic characteristics of Mongolian horses during walk, slow trot, and fast trot, and to explore potential associations between selected kinematic variables and race-performance grouping in a 12 km endurance race.

## 2. Materials and Methods

### 2.1. Selection of Horses

The experiment was conducted at the horse farm of the Vocational and Technical College of Inner Mongolia Agricultural University in November 2024, July 2025, and February 2026. Forty-six adult Mongolian horses (age 7.0 ± 1.5 years, weight 480 ± 50 kg) were selected by trainers with over 10 years of experience in Mongolian horse husbandry and gait training. All horses were in good health and met endurance race eligibility standards, comprising 28 stallions and 18 mares. Prior to the 12 km endurance race, the test horses underwent further examinations of body temperature, resting heart rate, and respiratory rate. Their behavioural states were observed for abnormalities and lameness to ensure all competing horses met physical condition standards. After the race, the top 23 Mongolian horses were classified into the excellent group based on their race results, while the remaining 23 were classified into the ordinary group. Because this classification relied on the result of one race only, it may have been affected by race-day condition, rider-related variation, and other contextual factors. In addition, the horses were not ridden by the same rider. Although all riders were experienced and all horses competed under identical race rules and course conditions, the potential influence of rider-related effects on race outcomes could not be completely ruled out.

### 2.2. Marking Key Points

**Figure 1:**
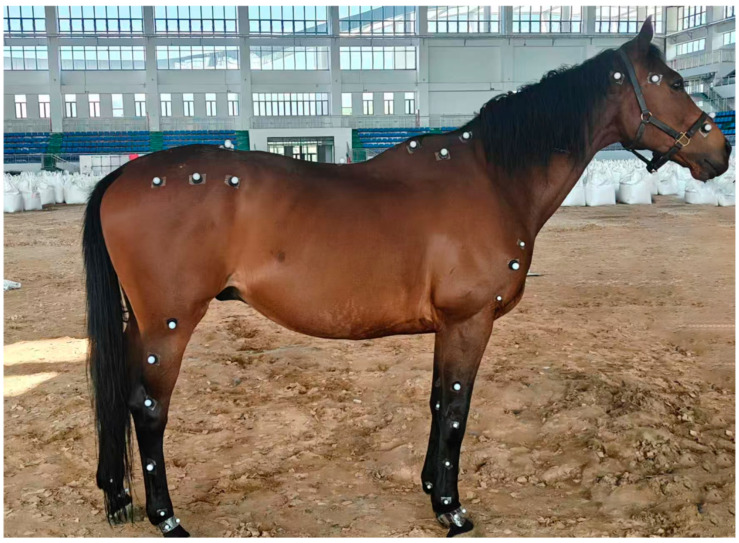
Mongolian horse with spherical reflective markers affixed at key anatomical points. The marker is a spherical reflector with a diameter of 16 mm, and all marker points are accurately pasted by the same veterinarian according to the anatomical position of Mongolian horses.

Spherical reflective markers (16 mm diameter) were attached to predefined anatomical landmarks on each Mongolian horse (Figure 1). To ensure consistent marker placement, all landmarks were identified and marked by the same veterinarian. Before attachment, the hair at each landmark was clipped to reduce skin-motion artefacts, and the coat and hooves were brushed to remove dirt and improve adhesion.

**Figure 2:**
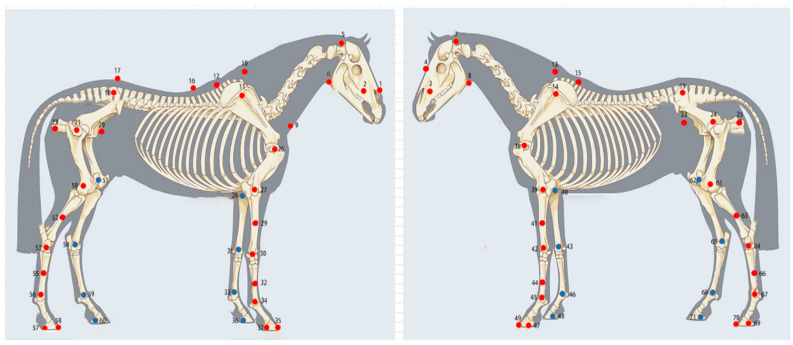
Anatomical locations of 71 key reflective markers on the Mongolian horse body. This includes 8 on the head, 17 on the trunk, 24 on the forelimbs, and 22 on the hindlimbs.

To capture overall body shape and limb motion, we defined 71 anatomical landmarks as marker locations based on established equine anatomy. These included 8 points on the head, 17 on the trunk, 24 on the forelimbs, and 22 on the hindlimbs [21] (Figure 2). Detailed marker definitions and locations are provided in Table 1.

### 2.3. Data Collection

**Figure 3:**
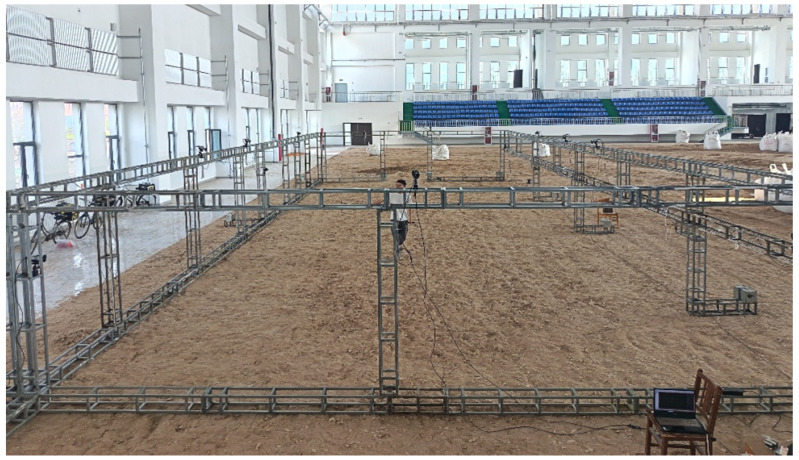
Acquisition Platform. All cameras are fixed by a 2.8 m-high truss and undergo system calibration using a T-shaped calibration wand before formal collection. The sampling frequency is uniformly set to 240 Hz.

Optical motion-capture trials were performed in an indoor riding arena with a sand–synthetic fibre surface. A synchronized recording setup was used, comprising 12 Qualisys infrared motion-capture cameras and two colour video cameras. To ensure stable marker tracking and adequate capture coverage, a closed capture volume was established within the arena according to the manufacturer’s guidelines (Figure 3).

**Figure 4:**
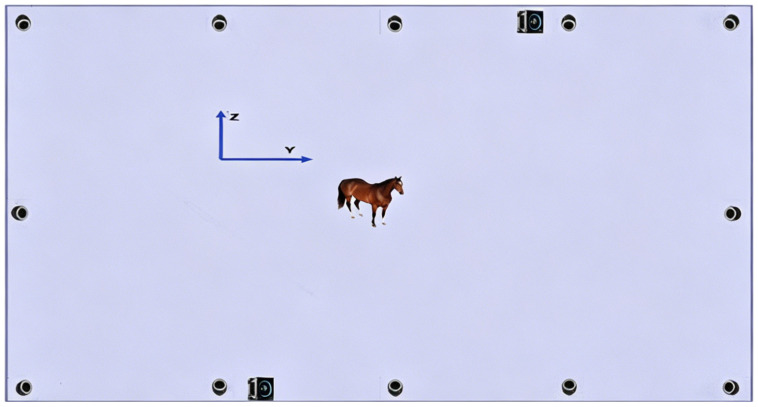
Camera Positioning Layout. Twelve infrared motion-capture cameras were arranged around the capture area to provide complementary viewing angles, and two colour video cameras were positioned along the two long sides.

A 10 m × 20 m rectangular capture area was built using a truss framework, with the cameras mounted at a height of 2.8 m. The camera layout is shown in Figure 4. This setup was designed to ensure broad field-of-view coverage and complementary viewing angles, while minimizing interference from horse movement.

Prior to formal data collection, the caretaker led each horse into the capture area for five consecutive days to allow free movement and acclimation. On the test day, horses were guided into the capture zone and recorded while performing three gaits in a fixed order (walk, slow trot, fast trot). A 2 min standing rest was provided between gait conditions. Total recording time per horse did not exceed 20 min, and sessions were avoided during fasting periods or high ambient temperatures to reduce fatigue and stress. All recordings for each horse were completed within a single day. Because some horses occasionally deviated from the intended gait (e.g., transitioning from walk to trot despite walk commands), gait labels were verified during post-processing by cross-referencing synchronized colour video, and segments inconsistent with the caretaker’s commands were excluded. Motion-capture data were sampled at 240 Hz.

### 2.4. Data Processing

**Figure 5:**
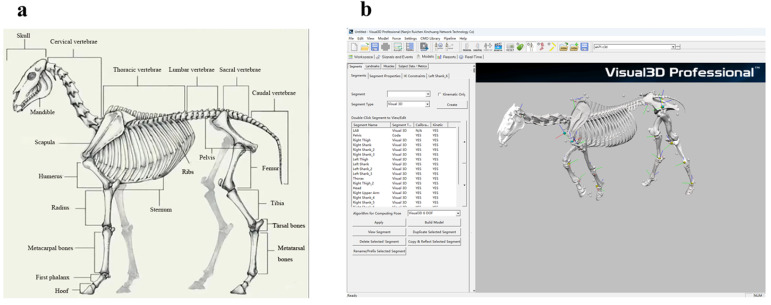
Skeletal Model of a Mongolian Horse. (**a**) Names of Mongolian Horse Skeletal Structures. (**b**) The Mongolian horse model constructed in Visual3D.

In motion-capture studies, kinematic parameters are commonly obtained by fitting skeletal models to marker trajectories; however, this approach is still uncommon in equine research. In the present study, we generated a three-dimensional skeletal model of a Mongolian horse using a handheld 3D scanner. The model comprised 29 major bones, including the cranium, vertebral segments, pelvis, femur, tibia, and tarsal bones (Figure 5). The reconstructed skeletal geometry was imported into Blender (v4.3) for model assembly and visualization [22].

The reconstructed skeleton was exported in OBJ/MTL format and imported into Visual3D. Based on Mongolian horse anatomy, functionally related bones were grouped into major segments using the skeletal modelling tool. Specifically, the skull and mandible were combined as the head segment; the thoracic vertebrae, ribs, sternum, and scapulae were combined as the trunk segment; and the lumbar vertebrae, sacrum, and coccygeal vertebrae were combined as the tail segment. Limb bones were retained as individual segments [23].

To build the segment geometries in Visual3D, each bone was approximated using simple shapes defined by the marker landmarks. For example, the radius was modelled as a frustum whose length and end diameters were determined from markers 11 and 14. Using the same approach, the skull, spine, and limb segments were constructed according to their anatomical connections. Segment parameters (e.g., length, orientation, and joint-centre locations) were iteratively adjusted to match the morphology of a Mongolian horse. In Visual3D, the mass properties of each segment were specified, and segment centres of mass were computed using the Calculate from Geometry function based on the reconstructed segment geometry [24]. Mongolian horses are a small grassland breed that has undergone long-term selection for endurance; compared with Thoroughbred and Warmblood horses, they are reported to have relatively more robust limb bones and a lower centre of mass, which may contribute to stable locomotion [25].

Using the procedures described above, we reconstructed a complete skeletal model of the Mongolian horse (Figure 5). Kinematic features were then computed from this model in Visual3D using a custom script, yielding nine kinematic parameters (Table 2).

## 3. Data Analysis

A total of 46 adult Mongolian horses (*n* = 46) were included in this study and were divided into an excellent group (*n* = 23, top 23 finishers) and an ordinary group (*n* = 23, the remaining 23 horses) based on their race results. The baseline characteristics of the two groups of horses are as follows: the gender distribution was 15 male and 8 female in the excellent group, and 13 male and 10 female in the ordinary group; the age distribution was 7.2 years in the excellent group and 6.8 years in the ordinary group; the weight distribution was 485 kg in the excellent group and 462 kg in the ordinary group. Previous work suggests that equine joint angles show limited associations with age, body weight, or BCS; therefore, these variables were not treated as primary confounders in the present analysis [26].

Stride length was defined as the distance between two successive ground contacts of the same limb. For gait-cycle segmentation, we used the periodic variation in the distance between markers 58 and 35 as a surrogate signal because it showed consistent maxima and minima across stride cycles, thereby facilitating identification of cycle boundaries. This cyclical pattern enabled accurate segmentation of the gait cycle and extraction of kinematic parameters. Before analysis, strides with marker loss, obvious tracking artefacts, or gait labels inconsistent with synchronized video review were excluded. After quality control, 20 valid gait cycles were retained for each gait in each horse.

Descriptive statistical analyses of all kinematic variables during walk, slow trot, and fast trot were performed using SPSS 27.0. For each variable, the mean and standard deviation were calculated, and the results are presented as mean ± SD. Because the biomechanical characteristics and measurement scales of the kinematic variables differed across gait conditions, and because most variables were closely related to speed, separate linear mixed-effects models were fitted for each gait. In each model, horse identity was included as a random effect to account for repeated stride measurements within horses, whereas performance group and speed were treated as fixed effects. Model assumptions were evaluated by visual inspection of residual-versus-fitted plots and quantile–quantile plots of residuals to assess homoscedasticity and residual normality. The presence of influential outlying observations was also checked. Because data collection was performed in three recording periods (November 2024, July 2025, and February 2026), potential recording-period effects cannot be excluded and should be considered when interpreting the results.

### 3.1. Kinematic Differences During Walk

During walk, Tr, Hr, Er, and MaxRf were all higher in the excellent group than in the ordinary group. The corresponding values were 43.11 ± 4.02° versus 40.26 ± 3.68° for Tr, 26.07 ± 2.45° versus 23.88 ± 2.31° for Hr, 52.15 ± 3.12° versus 50.07 ± 2.98° for Er, and 103.71 ± 3.28° versus 100.43 ± 3.15° for MaxRf. The estimated differences were 2.86°, 2.18°, 2.07°, and 3.24°, respectively, with corresponding 95% confidence intervals of [0.92, 4.78], [0.85, 3.53], [0.61, 3.55], and [1.56, 4.82]. All four variables differed significantly between groups (*p* < 0.001).

In contrast, no significant between-group differences were observed for Stl, MaxRh, MinPh, or MinPf. The Stl values were 171.80 ± 8.25 cm in the excellent group and 166.73 ± 9.91 cm in the ordinary group, with an estimated difference of 5.19 cm (95% CI: [−0.18, 10.30], *p* = 0.17). The MaxRh values were 110.92 ± 3.65° and 107.87 ± 3.42°, respectively, with an estimated difference of 2.89° (95% CI: [−0.28, 5.82], *p* = 0.29). The MinPh values were 68.24 ± 2.71° and 66.55 ± 2.58°, respectively, with an estimated difference of 1.68° (95% CI: [−0.33, 3.41], *p* = 0.34). The MinPf values were 68.23 ± 2.64° in the excellent group and 66.15 ± 2.51° in the ordinary group, with an estimated difference of 2.21° (95% CI: [−0.12, 4.28], *p* = 0.065). Although MinPf showed a numerical trend toward a between-group difference, this difference did not reach statistical significance (Table 3).

### 3.2. Kinematic Differences During Slow Trot

During slow trot, the differences between the excellent group and the ordinary group were mainly observed in stride length, elbow joint range of motion, and forward-extension-related variables. The Stl value was significantly higher in the excellent group than in the ordinary group (223.51 ± 9.42 cm vs. 218.66 ± 8.87 cm; estimate = 4.62 cm, 95% CI [1.23, 8.27], *p* = 0.009). Similarly, the Er value was significantly higher in the excellent group than in the ordinary group (56.63 ± 3.48° vs. 53.37 ± 3.25°; estimate = 3.16°, 95% CI [1.38, 5.14], *p* < 0.001).

For the forward-extension-related variables, both MinPh and MinPf were significantly higher in the excellent group than in the ordinary group. Specifically, MinPh was 69.48 ± 2.95° in the excellent group and 67.68 ± 2.81° in the ordinary group (estimate = 1.85°, 95% CI [0.15, 3.45], *p* = 0.033), while MinPf was 65.68 ± 2.76° in the excellent group and 63.21 ± 2.63° in the ordinary group (estimate = 2.41°, 95% CI [0.78, 4.16], *p* = 0.004).

In contrast, although Tr, Hr, MaxRh, and MaxRf were higher in the excellent group than in the ordinary group, none of these variables showed statistically significant between-group differences. The corresponding values were 58.41 ± 4.15° versus 56.74 ± 3.92° for Tr (estimate = 1.59°, 95% CI [−0.45, 3.79], *p* = 0.121), 27.49 ± 2.83° versus 26.64 ± 2.69° for Hr (estimate = 0.91°, 95% CI [−0.52, 2.22], *p* = 0.223), 112.39 ± 3.81° versus 108.86 ± 3.57° for MaxRh (estimate = 3.14°, 95% CI [−0.46, 6.84], *p* = 0.21), and 102.39 ± 3.62° versus 99.24 ± 2.73° for MaxRf (estimate = 2.73°, 95% CI [−0.12, 5.48], *p* = 0.18). Overall, the significant differences observed during slow trot were mainly concentrated in stride length, elbow joint range of motion, and forelimb and hindlimb forward-extension angles (Table 4).

### 3.3. Kinematic Differences During Fast Trot

During fast trot, the between-group differences were more pronounced than those observed during walk and slow trot, with a greater number of variables showing statistically significant differences. The Stl value was significantly higher in the excellent group than in the ordinary group (293.73 ± 10.65 cm vs. 276.95 ± 10.12 cm; estimate = 16.64 cm, 95% CI [11.52, 21.34], *p* < 0.001).

Similarly, the Tr, Hr, and Er values were all significantly higher in the excellent group than in the ordinary group. Specifically, Tr was 65.21 ± 4.58° in the excellent group and 60.93 ± 4.32° in the ordinary group (estimate = 4.18°, 95% CI [1.85, 6.79], *p* < 0.001), Hr was 30.43 ± 3.12° and 28.62 ± 2.98°, respectively (estimate = 1.61°, 95% CI [0.35, 3.27], *p* = 0.015), and Er was 60.81 ± 3.75° and 58.80 ± 3.51°, respectively (estimate = 1.97°, 95% CI [0.42, 3.60], *p* = 0.014).

For the hindlimb retraction- and forelimb forward-extension-related variables, both MaxRh and MinPf were significantly higher in the excellent group than in the ordinary group. MaxRh was 114.12 ± 4.02° in the excellent group and 110.71 ± 3.85° in the ordinary group (estimate = 3.31°, 95% CI [1.38, 5.44], *p* = 0.001), while MinPf was 63.43 ± 2.89° in the excellent group and 61.86 ± 2.76° in the ordinary group (estimate = 1.67°, 95% CI [0.19, 3.35], *p* = 0.026).

In contrast, no statistically significant between-group differences were detected for MinPh or MaxRf. The MinPh values were 71.38 ± 3.15° in the excellent group and 69.72 ± 2.99° in the ordinary group, with an estimated difference of 1.48° (95% CI [−0.12, 3.44], *p* = 0.18). The MaxRf values were 101.18 ± 3.89° in the excellent group and 97.17 ± 3.72° in the ordinary group, with an estimated difference of 3.31° (95% CI [−0.22, 6.77], *p* = 0.24). Overall, during fast trot, the significant between-group differences were mainly observed in stride length, selected joint range-of-motion variables, and hindlimb retraction/forelimb forward-extension parameters (Table 5).

## 4. Discussion

Using optical motion capture and three-dimensional skeletal modelling, this pilot study quantified the kinematic characteristics of Mongolian horses during walk, slow trot, and fast trot, and preliminarily examined gait-related variables that may be associated with differences in 12 km endurance-race performance grouping. Although motion-capture-based skeletal reconstruction has been widely used in human biomechanics, its application in equine research remains relatively limited because of anatomical differences and the large number of markers required for accurate modelling in horses. By providing preliminary quantitative phenotypic data for Mongolian horses, this study complements previous work that has focused mainly on molecular mechanisms and contributes to the still limited literature on gait-related characterization of this breed. Overall, the present approach provides an initial framework for future kinematic studies of endurance horses, although its potential value for performance evaluation, breed management, or training will require further validation in larger studies.

### 4.1. Kinematic Characteristics During Walk

Walk is the most basic and stable gait in Mongolian horses and may, to some extent, reflect long-term adaptation to free-range grazing, load carrying, and movement across grassland terrain. In the present study, Mongolian horses exhibited relatively conservative joint excursion and a stable gait pattern during walk, which is broadly consistent with the functional role of this gait as a low-speed locomotor mode with relatively low mechanical demands. Descriptively, this pattern may indicate an emphasis on support stability and locomotor economy rather than large limb excursions or strong propulsive output [27].

During walk, Tr, Hr, Er, and MaxRf were significantly higher in the excellent group than in the ordinary group, whereas Stl, MaxRh, MinPh, and MinPf did not differ significantly between groups. These results suggest that, under walking conditions, group differences were more evident in selected joint-motion and forelimb movement variables than in stride length. A possible explanation is that horses in the excellent group may show subtle advantages in joint coordination and limb control during low-speed locomotion. However, such an interpretation should be made cautiously. Given the relatively low mechanical demand of walk, the observed differences may reflect variation in basic gait regulation rather than direct determinants of endurance performance. Accordingly, although several joint-related variables and forelimb retraction were higher in the excellent group, these parameters should not yet be considered robust or generalizable indicators of endurance ability without further validation [28].

### 4.2. Kinematic Characteristics During Slow Trot

As speed increased from walk to slow trot, stride length and selected joint excursions increased, while overall locomotor stability appeared to be maintained. Compared with some speed-oriented breeds, Mongolian horses showed a relatively moderate magnitude of kinematic change during slow trot, which may reflect a locomotor pattern favouring sustained progression while preserving stability. However, such cross-breed comparisons should be interpreted cautiously because methodological and experimental differences may limit direct comparability [29].

In the present study, Stl, Er, MinPh, and MinPf were significantly higher in the excellent group than in the ordinary group during slow trot, whereas Tr, Hr, MaxRh, and MaxRf did not differ significantly between groups. These findings suggest that stride length, elbow joint range of motion, and selected forelimb and hindlimb forward-extension variables may be the kinematic features most clearly distinguishing the two groups during slow trot. One possible explanation is that horses in the excellent group showed more effective step expansion and forward limb excursion under moderate-speed conditions. Nevertheless, these findings should be interpreted cautiously. The present results indicate statistical associations within this sample, but do not establish causality or confirm that these variables have stable discriminative value across broader Mongolian horse populations. Therefore, the slow trot findings are better regarded as preliminary evidence warranting further investigation rather than definitive evidence of performance-related locomotor mechanisms [30].

### 4.3. Kinematic Characteristics During Fast Trot

Among the three gaits examined, fast trot showed the greatest number of significant between-group differences. As speed increased further, stride length and several joint-motion variables also increased, suggesting that this gait placed greater demands on propulsion, interlimb coordination, and movement control. Under these conditions, Stl, Tr, Hr, Er, MaxRh, and MinPf were all significantly higher in the excellent group than in the ordinary group, whereas MinPh and MaxRf did not differ significantly between groups.

These findings suggest that, under the present experimental conditions, fast trot may be the gait in which differences between the excellent group and the ordinary group were most clearly expressed. In particular, the relatively large between-group difference in stride length may indicate that horses in the excellent group were better able to maintain greater forward displacement per stride at higher speeds. Similarly, the higher values observed for hip and elbow joint range of motion and maximum hindlimb retraction may suggest a greater contribution of coordinated limb movement and hindlimb propulsion during fast trot. However, these interpretations should be treated cautiously. The present results indicate statistical associations rather than causal relationships, and they do not demonstrate that greater stride length or larger joint excursions necessarily confer superior endurance performance. Therefore, these findings are better regarded as preliminary evidence of gait-related differences between groups under the specific conditions of this study [31,32,33].

### 4.4. Limitations of Research Methods and Future Prospects

This study has several limitations. First, the sample size was relatively modest (*n* = 46), and all horses were recruited from a single setting, which may limit the generalizability of the findings. Second, horse classification was based on the result of a single 12 km endurance race and therefore may not fully reflect long-term endurance capacity. Moreover, because the horses were not ridden by the same rider, rider-related effects could not be fully excluded, although all riders were experienced and all horses competed under the same race rules and course conditions. Third, the analyses were restricted to locomotion recorded on a flat indoor surface, and gait adaptations under uneven or more challenging terrains were not assessed. Fourth, the study focused exclusively on kinematic variables and did not include concurrent physiological or biomechanical indicators, such as electromyography, heart rate, or metabolic markers. Therefore, the functional implications of the observed gait differences remain unclear.

Future studies should include larger and more diverse cohorts of Mongolian horses from different farms and training backgrounds, examine locomotion under more ecologically relevant conditions, and integrate kinematic analysis with physiological, biomechanical, and biochemical measurements. Such efforts will be important for validating the present findings and for clarifying the potential relevance of these variables to performance evaluation in Mongolian horses [34].

## 5. Conclusions

This exploratory study preliminarily characterized the kinematics of Mongolian horses during walk, slow trot, and fast trot using optical motion capture and three-dimensional skeletal modelling. The results showed that the variables differentiating the excellent group from the ordinary group varied across gaits. Significant between-group differences were mainly observed in selected joint range-of-motion variables, stride length, and limb retraction/protraction parameters, depending on gait.

Overall, these findings provide preliminary evidence that gait-related kinematic differences may exist between performance groups in Mongolian horses. However, because this was an exploratory study with important methodological limitations, the results should be interpreted cautiously. Any practical implications for performance evaluation or training management remain tentative and require confirmation in larger studies.

## Figures and Tables

**Table 1:** Description and placement of marker setup on the horse’s body.

Number	Name of the Sticker	Location
1	Head-Front	Cranium-Rostral
2	Head-Right	Cranium-Right Parietal Region
3	Head-Left	Cranium-Left Basal Cranial Region
4	Head	Facial Region-Midline
5	Poll-Right	Poll-Right Lateral
6	Head-Right under	Poll-Right Ventrolateral
7	Poll-Left	Poll-Left Lateral
8	Head-Left under	Poll-Left Ventrolateral
9	Neck-low	Cervical Spine-Ventral Distal
10	Neck-top Left	Cervical Spine-Left Dorsal Proximal
11	Withers-right	Scapula-Right Spine of Scapula
12	Withers	Thoracic Spine-3rd Right Spinous Process
13	Neck-top Right	Cervical Spine-Right Dorsal Proximal
14	Withers-left	Scapula-Left Spine of Scapula
15	Back-1	Thoracic Spine-6th Left Spinous Process
16	Back-2	Thoracic Spine-10th Midline Spinous Process
17	Sacrum	Sacrum-Median Sacral Crest
18	Tuber-coxae Right	Coxal Bone-Right Tuber Coxae Distal
19	Tuber-coxae Left	Coxal Bone-Left Tuber Coxae Distal
20	Hip joint Right-Front	Hip Joint-Right Anterolateral
21	Hip joint Right	Hip Joint-Right Lateral
22	Hip joint Right-Behind	Hip Joint-Right Posterolateral
23	Hip joint Left-Front	Hip Joint-Left Anterolateral
24	Hip joint Left	Hip Joint-Left Lateral
25	Hip joint Left-Behind	Hip Joint-Left Posterolateral
26	Right FrontOut-Top	Humerus-Right Greater Tubercle Distal
27	Right FrontIn-Upper	Elbow Joint-Right Medial Proximal Joint Space
28	Left FrontIn-Upper	Elbow Joint-Left Medial Proximal Joint Space
29	Right Radius	Radius-Right Middle Shaft
30	Right FrontOut-Knee	Carpal Joint-Right Lateral
31	Left FrontIn-Knee	Carpal Joint-Left Medial
32	Right Metacarpal bone	Metacarpal Bone III-Right Midshaft
33	Left FrontIn-Fetlock	Carpal Joint-Left Medial Collateral Ligament Proximal
34	Right FrontOut-Fetlock	Carpal Joint-Right Lateral Collateral Ligament Proximal
35	Right FrontOut-Mid	Hoof-Right Dorsolateral Proximal Hoof Wall
36	Left FrontIn-Hoof	Hoof-Left Dorsal Proximal Hoof Wall
37	Right FrontOut-Hoof	Hoof-Right Proximal Hoof Bulb
38	Left FrontOut-Top	Humerus-Left Greater Tubercle Distal
39	Left FrontOut-Upper	Elbow Joint-Left Lateral Proximal Joint Space
40	Right FrontIn-Upper	Elbow Joint-Right Lateral Proximal Joint Space
41	Left Radius	Radius-Left Middle Shaft
42	Left FrontOut-Knee	Carpal Joint-Left Lateral
43	Right FrontIn-Knee	Carpal Joint-Right Medial
44	Left Metacarpal bone	Metacarpal Bone III-Left Midshaft
45	Left FrontOut-Fetlock	Carpal Joint-Left Lateral Collateral Ligament Proximal
46	Right FrontIn-Fetlock	Carpal Joint-Right Medial Collateral Ligament Proximal
47	Left FrontOut-Hoof	Hoof-Left Proximal Hoof Bulb
48	Left FrontOut-Mid	Hoof-Left Dorsolateral Proximal Hoof Wall
49	Right FrontIn-Hoof	Hoof-Right Dorsal Proximal Hoof Wall
50	Right BackOut-Upper	Tarsal Joint-Right Lateral Collateral Ligament Proximal
51	Left BackIn-Upper	Tarsal Joint-Left Medial Collateral Ligament Proximal
52	Right Tibia	Tibia-Right Middle Shaft
53	Right BackOut-Hock	Fibula-Right Lateral Shaft
54	Left BackIn-Hock	Fibula-Left Medial Shaft
55	Right Metatarsal bone	Metatarsal Bone III-Right Midshaft
56	Right BackOut-Ergot	Tarsometatarsal Joint-Right Lateral Proximal Joint Space
57	Right BackOut-Heel	Hoof-Right Plantar Proximal Hoof Bulb
58	Right BackOut-Mid	Hoof-Right Lateroplantar Proximal Hoof Wall
59	Left BackIn-Ergot	Tarsometatarsal Joint-Left Medial Proximal Joint Space
60	Left BackIn-Heel	Hoof-Left Plantar Proximal Hoof Wall
61	Left BackOut-Upper	Tarsal Joint-Left Lateral Collateral Ligament Proximal
62	Right BackIn-Upper	Tarsal Joint-Right Medial Collateral Ligament Proximal
63	Left Tibia	Tibia-Left Middle Shaft
64	Left BackOut-Hock	Fibula-Left Lateral Shaft
65	Right BackIn-Hock	Fibula-Right Medial Shaft
66	Left Metatarsal bone	Metatarsal Bone III-Left Midshaft
67	Left BackOut-Ergot	Tarsometatarsal Joint-Left Lateral Proximal Joint Space
68	Right BackIn-Ergot	Tarsometatarsal Joint-Right Medial Proximal Joint Space
69	Right BackOut-Heel	Hoof-Right Plantar Distal Hoof Bulb
70	Right BackOut-Mid	Hoof-Right Lateroplantar Distal Hoof Wall
71	Left BackIn-Heel	Hoof-Left Plantar Distal Hoof Wall

**Table 2:** Kinematic parameters of Mongolian Horses.

Parameters	Abbreviation	Introduction
1. Speed (m/s)	Sp	The average speed of a horse over a single gait cycle
2. Stride (cm)	Stl	The horizontal distance between the two consecutive points of contact for the same front hoof
3. Range of motion of the tarsal joint (°)	Tr	The difference between the maximum and minimum angles of the tarsal joint (50, 53, 56)
4. Range of motion of the hip joint (°)	Hr	The difference between the maximum and minimum angles of the hip joint (18, 21, 50)
5. Range of motion of the elbow joint (°)	Er	The difference between the maximum and minimum angles of the elbow joint (26, 27, 30)
6. Maximum rear limb retraction angle (°)	MaxRh	The maximum angle formed by the line connecting points 21 and 57 with the horizontal line
7. Minimum forward extension angle of the hind limbs (°)	MinPh	The minimum angle formed by the line connecting points 21 and 57 with the horizontal line
8. Maximum retraction angle of the forelimb (°)	MaxRf	The maximum angle formed by the line connecting points 11 and 32 with the horizontal line
9. Minimum forward extension angle of the forelimb (°)	MinPf	The minimum angle formed by the line connecting points 11 and 32 with the horizontal line

**Table 3:** Intergroup Difference Analysis of Kinematic Characteristics of Mongolian Horses during walk (Excellent group *n* = 23, Ordinary group *n* = 23).

Characteristics	Excellent Group	Ordinary Group	Estimate	95% CI	*p*-Value
Stl (cm)	171.80 ± 8.25	166.73 ± 9.91	5.19	[−0.18, 10.30]	0.17
Tr (°)	43.11 ± 4.02	40.26 ± 3.68	2.86	[0.92, 4.78]	<0.001
Hr (°)	26.07 ± 2.45	23.88 ± 2.31	2.18	[0.85, 3.53]	<0.001
Er (°)	52.15 ± 3.12	50.07 ± 2.98	2.07	[0.61, 3.55]	<0.001
MaxRh (°)	110.92 ± 3.65	107.87 ± 3.42	2.89	[−0.28, 5.82]	0.29
MinPh (°)	68.24 ± 2.71	66.55 ± 2.58	1.68	[−0.33, 3.41]	0.34
MaxRf (°)	103.71 ± 3.28	100.43 ± 3.15	3.04	[1.56, 4.82]	<0.001
MinPf (°)	68.23 ± 2.64	66.15 ± 2.51	2.21	[−0.12, 4.28]	0.065

Note: Values are presented as mean ± SD. Estimate represents the speed-adjusted estimated difference between the excellent group and the ordinary group derived from the gait-specific linear mixed-effects model.

**Table 4:** Intergroup Difference Analysis of Kinematic Characteristics of Mongolian Horses during slow trot (Excellent group *n* = 23, Ordinary group *n* = 23).

Characteristics	Excellent Group	Ordinary Group	Estimate	95% CI	*p*-Value
Stl (cm)	223.51 ± 9.42	218.66 ± 8.87	4.62	[1.23, 8.27]	0.009
Tr (°)	58.41 ± 4.15	56.74 ± 3.92	1.59	[−0.45, 3.79]	0.121
Hr (°)	27.49 ± 2.83	26.64 ± 2.69	0.91	[−0.52, 2.22]	0.223
Er (°)	56.63 ± 3.48	53.37 ± 3.25	3.16	[1.38, 5.14]	<0.001
MaxRh (°)	112.39 ± 3.81	108.86 ± 3.57	3.14	[−0.46, 6.84]	0.21
MinPh (°)	69.48 ± 2.95	67.68 ± 2.81	1.85	[0.15, 3.45]	0.033
MaxRf (°)	102.39 ± 3.62	99.24 ± 3.48	2.73	[−0.12, 5.48]	0.18
MinPf (°)	65.68 ± 2.76	63.21 ± 2.63	2.41	[0.78, 4.16]	0.004

Note: Values are presented as mean ± SD. Estimate represents the speed-adjusted estimated difference between the excellent group and the ordinary group derived from the gait-specific linear mixed-effects model.

**Table 5:** Intergroup Difference Analysis of Kinematic Characteristics of Mongolian Horses during fast trot (Excellent group *n* = 23, Ordinary group *n* = 23).

Characteristics	Excellent Group	Ordinary Group	Estimate	95% CI	*p*-Value
Stl (cm)	293.73 ± 10.65	276.95 ± 10.12	16.64	[11.52, 21.34]	<0.001
Tr (°)	65.21 ± 4.58	60.93 ± 4.32	4.18	[1.85, 6.79]	<0.001
Hr (°)	30.43 ± 3.12	28.62 ± 2.98	1.61	[0.35, 3.27]	0.015
Er (°)	60.81 ± 3.75	58.80 ± 3.51	1.97	[0.42, 3.60]	0.014
MaxRh (°)	114.12 ± 4.02	110.71 ± 3.85	3.31	[1.38, 5.44]	0.001
MinPh (°)	71.38 ± 3.15	69.72 ± 2.99	1.48	[−0.12, 3.44]	0.18
MaxRf (°)	101.18 ± 3.89	97.17 ± 3.72	3.31	[−0.22, 6.77]	0.24
MinPf (°)	63.43 ± 2.89	61.86 ± 2.76	1.67	[0.19, 3.35]	0.026

Note: Values are presented as mean ± SD. Estimate represents the speed-adjusted estimated difference between the excellent group and the ordinary group derived from the gait-specific linear mixed-effects model.

## Data Availability

The data presented in this study are available on request from the corresponding author due to the fact that the data will be utilized in subsequent related research projects. To ensure the consistency and completeness of follow-up studies, we are temporarily unable to make the data publicly available at this stage. Researchers interested in accessing the data may contact the corresponding author directly, and we will provide relevant support and access arrangements in compliance with ethical and privacy protection requirements after completing the follow-up research.
